# Impact of Antithrombotic Therapy on Thrombotic and Bleeding Complications after Elective Endovascular Repair of Abdominal Aortic Aneurysms

**DOI:** 10.1007/s00270-024-03946-z

**Published:** 2025-01-16

**Authors:** Josephine Kranendonk, Ad A. Vermulst, Daphne van der Veen, Cornelis Kramers, Michiel C. Warlé, Michel M. P. J. Reijnen

**Affiliations:** 1 Department of Surgery, Radboud University Medical Center, Route 618, Geert Grooteplein Zuid 10, 6525 GA Nijmegen, The Netherlands; 2 Geestelijke Gezondheidszorg (Mental Health Care) Oost-Brabant, Boekel, The Netherlands; 3 Department of Surgery, Rijnstate Hospital, Arnhem, The Netherlands; 4 Department of Internal Medicine and Pharmacy, Radboud University Medical Center, Nijmegen, The Netherlands; 5 Department of Clinical Pharmacy, Canisius Wilhelmina Hospital, Nijmegen, The Netherlands; 6 Multimodality Medical Imaging Group, Faculty of Science and Technology, University of Twente, Enschede, The Netherlands

**Keywords:** Endovascular aneurysm repair (EVAR), Antithrombotic therapy, MACE, Prosthetic limb occlusion, Thrombosis

## Abstract

**Purpose:**

To investigate the influence of antithrombotic therapy on occurrence of thrombotic and bleeding complications after endovascular aneurysm repair (EVAR).

**Methods:**

In this retrospective single-center cohort study, patients who underwent elective endovascular aneurysm repair for abdominal aortic aneurysm were categorized into three antithrombotic groups: single antiplatelet therapy (SAPT), anticoagulants, or dual antiplatelet therapy (DAPT). Outcome measures were the incidence of major adverse cardiovascular events (MACE), prosthetic limb occlusions, and bleeding complications during follow-up.

**Results:**

Among 616 patients (SAPT: *n* = 450, anticoagulants: *n* = 84, and DAPT: *n* = 82), Kaplan–Meier analysis showed no significant difference (log-rank *p* = 0.37) in incidence of MACE between patients receiving SAPT (20.9%), anticoagulants (25.0%), and DAPT (14.6%) during a median follow-up of almost 4 years. In multivariable Cox regression analysis, only age (HR = 1.03; 95% CI 1.01–1.06, *p* = 0.01) and American Society of Anesthesiologists (ASA) classification (HR = 1.46; 95% CI 1.12–1.91; *p* = 0.01) were significant predictors for MACE. Prosthetic limb occlusion was observed in 38 patients during a median follow-up of 4 years; incidence between patients receiving SAPT (5.8%), anticoagulants (10.7%), and DAPT (3.7%) was not significantly different (log-rank *p* = 0.08). Age (HR = 0.96; 95% CI 0.92–1.00; *p* = 0.03) and use of anticoagulants (HR = 3.79, 95% CI 1.46–9.83; *p* < 0.01) were significant predictors for prosthetic limb occlusion. Bleeding complications occurred in 73 patients during median follow-up of almost 4 years, without significant difference (log rank *p* = 0.06) in incidence between patients receiving SAPT (10.7%), anticoagulants (19.0%), and DAPT (11.0%). ASA classification (HR = 1.74; 95% CI 1.23–2.46; *p* < 0.01) was a significant predictor for bleeding complications.

**Conclusion:**

Use of anticoagulants after EVAR appears to be associated with a higher risk of prosthetic limb occlusion compared to the use of single or dual antiplatelet therapy.

**Graphical Abstract:**

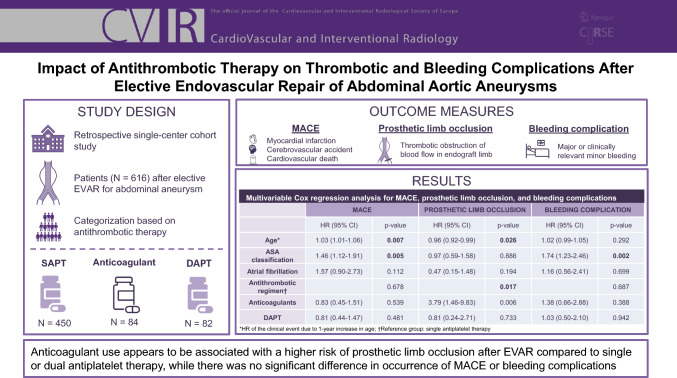

**Supplementary Information:**

The online version contains supplementary material available at 10.1007/s00270-024-03946-z.

## Introduction

Endovascular aortic repair (EVAR) has become a broadly accepted approach in the management of patients with abdominal aortic aneurysms (AAA) with suitable anatomy [1]. EVAR is associated with lower perioperative mortality and morbidity compared to open surgical repair, and long-term outcomes have significantly improved with newer generations of stent grafts [2–11].

Advancements in technology and procedural techniques, in combination with increased expertise, have substantially enhanced the safety and durability of EVAR, establishing it as the preferred treatment modality for the majority of patients with AAA [12, 13]. Related to its minimal invasive character, EVAR has been increasingly offered to the older population that is characterized by considerable comorbidity [14, 15].

Although the advantages of EVAR are clear, postprocedural thrombotic complications pose a substantial risk to patients’ health. Common thrombotic complications after EVAR are major adverse cardiovascular events, such as acute myocardial infarction or cerebrovascular accidents, as well as prosthetic limb occlusions [16–19]. The European Society for Vascular Surgery (ESVS) guidelines therefore recommend lifelong single antiplatelet therapy (SAPT) after EVAR procedures since its use is known to improve long-term outcomes, mainly by reducing the risk of thrombotic cardiovascular events [20–23].

Insights from studies concerning patients with peripheral arterial disease (PAD) and coronary artery disease (CAD) provide additional evidence on potential strategies for managing thrombotic risks in EVAR patients. SAPT will usually be intensified to dual antiplatelet therapy (DAPT) in patients with PAD following endovascular revascularization to improve stent patency according to the European Society of Cardiology (ESC) and ESVS guidelines [24, 25]. Furthermore, the Voyager PAD trial showed that adding a low-dose of the direct oral anticoagulant (DOAC) rivaroxaban to aspirin can significantly reduce thrombotic complications in PAD patients after lower-extremity revascularization. [26] Similarly, for patients with coronary artery disease (CAD), ESC guidelines recommend DAPT following percutaneous coronary intervention with stenting or in acute coronary syndrome. [27] In selected patients with stable CAD at high thrombotic risk, prolonged DAPT or low-dose rivaroxaban plus aspirin may offer additional benefit, as supported by the COMPASS trial. [28]

Given the increasing comorbidity of the EVAR population, many patients are already on anticoagulant therapy or dual antiplatelet therapy (DAPT) for indications such as atrial fibrillation or prior coronary or lower-limb interventions. The aim of this study was to evaluate the influence of these different antithrombotic regimens on thrombotic and bleeding outcomes after elective EVAR procedures for AAA.

## Methods

### Study Design

We conducted a retrospective, observational cohort study including patients who underwent an elective EVAR for AAA in a tertiary referral hospital from 2010 to 2020. This study was approved by the local Institutional Review Board (study number: 2022-2106). Studies involving the retrospective review, collection, and analysis of patient records do not fall under the Dutch Medical Research Involving Human Subjects Act (WMO), and therefore, individual patient informed consent was not required. The opt-out registry of the institute was consulted to find out whether patients had objected to participating in scientific research.

### Patient Population

Consecutive patients undergoing primary elective EVAR for AAA, including patients treated with fenestrated EVAR, were included. Patients were classified into three different antithrombotic groups based on their pharmacological treatment following the EVAR procedure. The single antiplatelet therapy (SAPT) group was treated with either aspirin or clopidogrel, the anticoagulants group was treated with either a vitamin K antagonist (VKA) or a direct oral anticoagulant (DOAC), and the dual antiplatelet therapy (DAPT) group was treated with aspirin in combination with a P2Y12 inhibitor. There were no patients who were concurrently using both anticoagulant and antiplatelet therapy. Perioperative management of antithrombotic therapy was done according to the European and Dutch guidelines on management of abdominal artery aneurysms [13, 29]. This implies that patients on SAPT could continue their medication without interruption to mitigate thrombotic and cardiac risks. Patients using VKAs or DOACs were discontinued a minimum of five days and two days before surgery, respectively, to minimize the potential for excessive bleeding. Depending on the indications necessitating their use, in some instances anticoagulation was bridged during the perioperative period using a low-molecular-weight heparin (LMWH). Treatment with VKA or DOAC was resumed 24 h after the procedure in case there were no signs of postprocedural bleeding complications. DAPT was transitioned to SAPT five to seven days before surgery, and patients resumed their DAPT the day after surgery. These strategies were adhered to in the absence of signs of postoperative bleeding, which could potentially have led to delayed resumption of the regular antithrombotic regimen in certain cases.

### Data Collection and Management

Perioperative and postoperative data were retrospectively derived from patients’ electronical medical records. Available background variables were obtained on the day of procedure and included age, sex, body mass index (BMI), American Society of Anesthesiologists (ASA) classification, smoking status, known risk factors for cardiovascular disease (CVD), history of atrial fibrillation, and endograft device type. Patients were in follow-up until there was a modification in antithrombotic therapy, occurrence of death, or until December 31st, 2021. Prior to data processing, the information was de-identified.

### Outcome Measures

The outcome measures of interest were the occurrence of thrombotic or bleeding complications. Thrombotic complications of interest included major adverse cardiovascular events (MACE) and prosthetic limb occlusions. MACE was defined as the composite of acute myocardial infarction, ischemic cerebrovascular accidents including a transient ischemic attack, or cardiovascular death. Prosthetic limb occlusion was defined as a thrombotic obstruction of blood flow in one or both endograft limbs. Visceral branch patency of patients treated with FEVAR was not included in this outcome. All patients in our study underwent computed tomography (CT) as the standard imaging modality during follow-up. All major and clinically relevant minor bleeding complications, as defined by the International Society on Thrombosis and Haemostasis criteria, were recorded [30, 31]. Clinically relevant minor bleeding complications included bleeding leading to hospitalization (including presentation to an acute care facility without an overnight stay), a physician guided medical or surgical treatment for bleeding, or bleeding leading to a change in antithrombotic treatment.

### Statistical Analysis

Categorical data were summarized with numbers and percentages, and continuous data with means and standard deviations. Differences in baseline characteristics between the three antithrombotic groups were tested with Chi-square test for nominal variables or Fisher’s exact test if expected cell frequencies were < 5, Kruskal–Wallis test for the ordinal variable ASA classification, and with one-way-ANOVA for continuous variables. When significant differences in baseline characteristics were identified, post hoc testing with Bonferroni correction was applied to identify the exact group differences. Kaplan–Meier (KM) analyses with log-rank tests were used to compare differences in time-to-event for MACE, prosthetic limb occlusion, and bleeding complications in the different antithrombotic groups. Multivariable Cox regression analyses, adjusted for age, ASA-classification, history of atrial fibrillation, and antithrombotic treatment regimen, were used to identify which of these baseline characteristics were significantly associated with each of the outcome measures. When patients did not experience an outcome event patients were censored at date of death, date of switch to a different antithrombotic regimen, or at the end date of follow-up on December 31st, 2021. For the statistical analyses, SPSS version 29 (IBM, Armonk, NY) and R version 4.3.2 (The R Foundation for Statistical Computing, Vienna, Austria) were used; *p* values of 0.05 or less were considered significant.

## Results

A total of 616 patients (SAPT: *n* = 450, anticoagulants: *n* = 84, and DAPT: *n* = 82) were included, with a mean age of 73.1 years, 85.9% was male. Baseline characteristics of the study population, stratified by antithrombotic regimen, are presented in Table 1. Post hoc testing with Bonferroni correction showed that patients in the anticoagulants group were significantly older (*p* = 0.042) and had a higher ASA classification (*p* < 0.001) than those in the SAPT group. There were no other statistically significant differences in distribution of age or ASA classification between the three groups. Risk factors for cardiovascular disease (CVD) were prevalent in 87.5% of the study population and included hypertension, diabetes mellitus, hyperlipidemia, or smoking. The incidence of atrial fibrillation was significantly higher (*p* < 0.001) among patients using anticoagulants compared to those on SAPT or DAPT. The Endurant endograft was used less frequently in patients using DAPT compared to those using SAPT, whereas the Anaconda endograft was used more frequently in patients using DAPT compared to those using SAPT. There were no other statistically significant differences in use of endografts between the three groups, nor did the use of endografts change over time. Known geometrical risk factors for prosthetic limb occlusion did not significantly differ between the different antithrombotic groups and included presence of severe iliac stenosis (defined as 30% or more luminal stenosis or a pressure gradient of 10 mmHg or more), severe iliac tortuosity, or kinking of the endograft.

**Table 1:** Baseline characteristics of the study population

	SAPT (*N* = 450)	Anticoagulants (*N* = 84)	DAPT (*N* = 82)	*p* value^†^
*Demographics*				
Age, mean (SD)	72.5 (8.3)	75.0 (8.0)	74.2 (8.1)	0.022
Male sex, No (%)	382 (84.9)	74 (88.1)	73 (89.0)	0.503
*Clinical characteristics*				
BMI, mean (SD)	26.6 (3.93)	27.5 (4.72)	26.9 (3.18)	0.107
ASA classification, No (%)				< 0.001
ASA 2	191 (42.4)	16 (19.0)	20 (24.4)	
ASA 3	203 (45.1)	46 (54.8)	55 (67.1)	
ASA 4	56 (12.4)	22 (26.2)	7 (8.5)	
Current smoker, No (%)	142 (35.9)	28 (39.4)	24 (30.8)	0.532
Risk factor CVD, No (%)*	390 (86.7)	77 (91.7)	72 (87.8)	0.444
Atrial fibrillation, No (%)	24 (5.6)	56 (70.9)	5 (6.4)	< 0.001
*Procedural characteristics*				
Device type, No (%)				0.009
Endurant	257 (57.1)	47 (56.0)	31 (37.8%)	
Excluder	125 (27.8)	19 (22.6)	30 (37.8)	
Anaconda	28 (6.2)	8 (9.5)	17 (20.7)	
Other endograft	40 (8.9)	10 (11.9)	4 (4.9)	
Risk factor prosthetic limb occlusion, No (%)**	8 (1.78%)	4 (4.76%)	2 (2.44%)	0.167

*SAPT* single antiplatelet therapy, *DAPT* dual antiplatelet therapy, *BMI*, body mass index, *ASA* American Society of Anesthesiologists, *CVD* cardiovascular disease

^*^Indicates the presence of one or more risk factors for cardiovascular disease; diabetes, hypertension, hyperlipidemia, smoking

^**^Indicates the presence of one or more risk factors for prosthetic limb occlusion; severe iliac stenoses (defined as 30% or more luminal stenosis or a pressure gradient of 10 mmHg or more), severe iliac tortuosity, or kinking of endograft

^†^Statistical tests: Chi-square (male sex, current smoker, risk factor CVD, atrial fibrillation, and device type), Fisher’s exact test (risk factor prosthetic limb occlusion), Kruskal–Wallis (ASA classification), and one-way ANOVA (age, BMI)

**Fig. 1:**
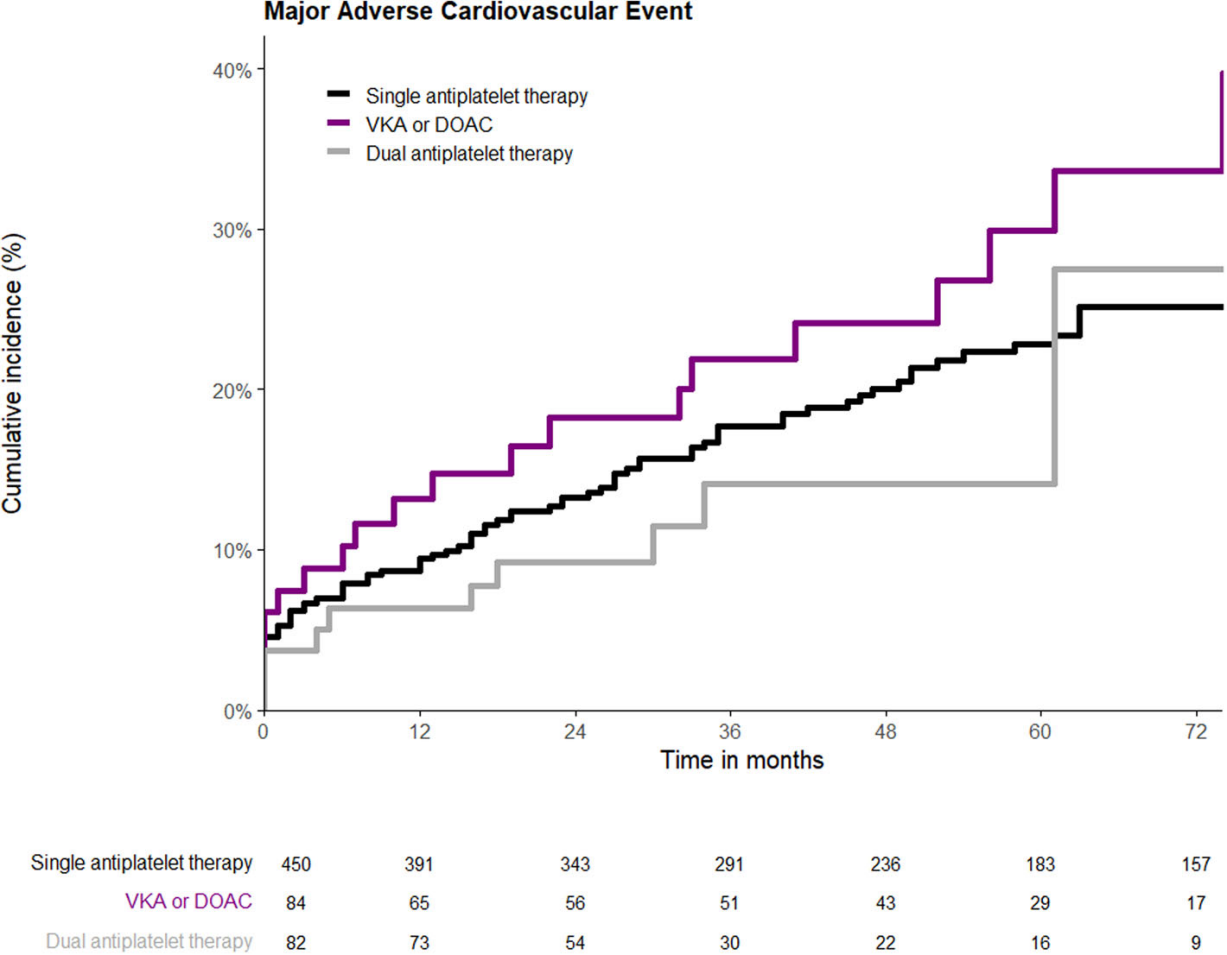
Kaplan–Meier time-to-event analysis for MACE based on antithrombotic regimen

A total of 165 thrombotic complications, including MACE and prosthetic limb occlusion, occurred during follow-up, along with 73 clinically relevant bleeding complications. During a median follow-up of 3 years and 10 months (IQR 4 years and 11 months) MACE was observed in a total of 127 patients (20.6%). Figure 1 shows the KM analysis for MACE in the different antithrombotic groups. The log-rank test indicated no statistically significant difference (*p* = 0.365) in the occurrence of MACE between patients receiving SAPT (20.9%), anticoagulants (25.0%), and DAPT (14.6%). Multivariable Cox regression analysis (Table 2) showed that age (HR = 1.03; 95% CI 1.01–1.06, *p* = 0.007) and ASA classification (HR = 1.46; 95% CI 1.12–1.91; *p* = 0.005) were significantly associated with MACE.

**Table 2:** Multivariable Cox regression analysis for major adverse cardiovascular events, prosthetic limb occlusion, and bleeding complications during follow-up

Variable	Major adverse cardiovascular events		Prosthetic limb occlusion		Bleeding complication	
	Hazard Ratio (95% CI)	*p* value	Hazard Ratio (95% CI)	*p* value	Hazard Ratio (95% CI)	*p* value
Age*	1.03 (1.01–1.06)	0.007	0.96 (0.92–0.99)	0.026	1.02 (0.99–1.05)	0.292
ASA classification	1.46 (1.12–1.91)	0.005	0.97 (0.59–1.58)	0.886	1.74 (1.23–2.46)	0.002
Atrial fibrillation	1.57 (0.90–2.73)	0.112	0.47 (0.15–1.48)	0.194	1.16 (0.56–2.41)	0.699
Antithrombotic regimen†		0.678		0.017		0.687
Anticoagulants	0.83 (0.45–1.51)	0.539	3.79 (1.46–9.83)	0.006	1.38 (0.66–2.88)	0.388
DAPT	0.81 (0.44–1.47)	0.481	0.81 (0.24–2.71)	0.733	1.03 (0.50–2.10)	0.942

*ASA* American Society of Anesthesiologists, *DAPT* dual antiplatelet therapy

^*^HR of the clinical event due to 1-year increase in age

^†^Reference group: single antiplatelet therapy

**Fig. 2:**
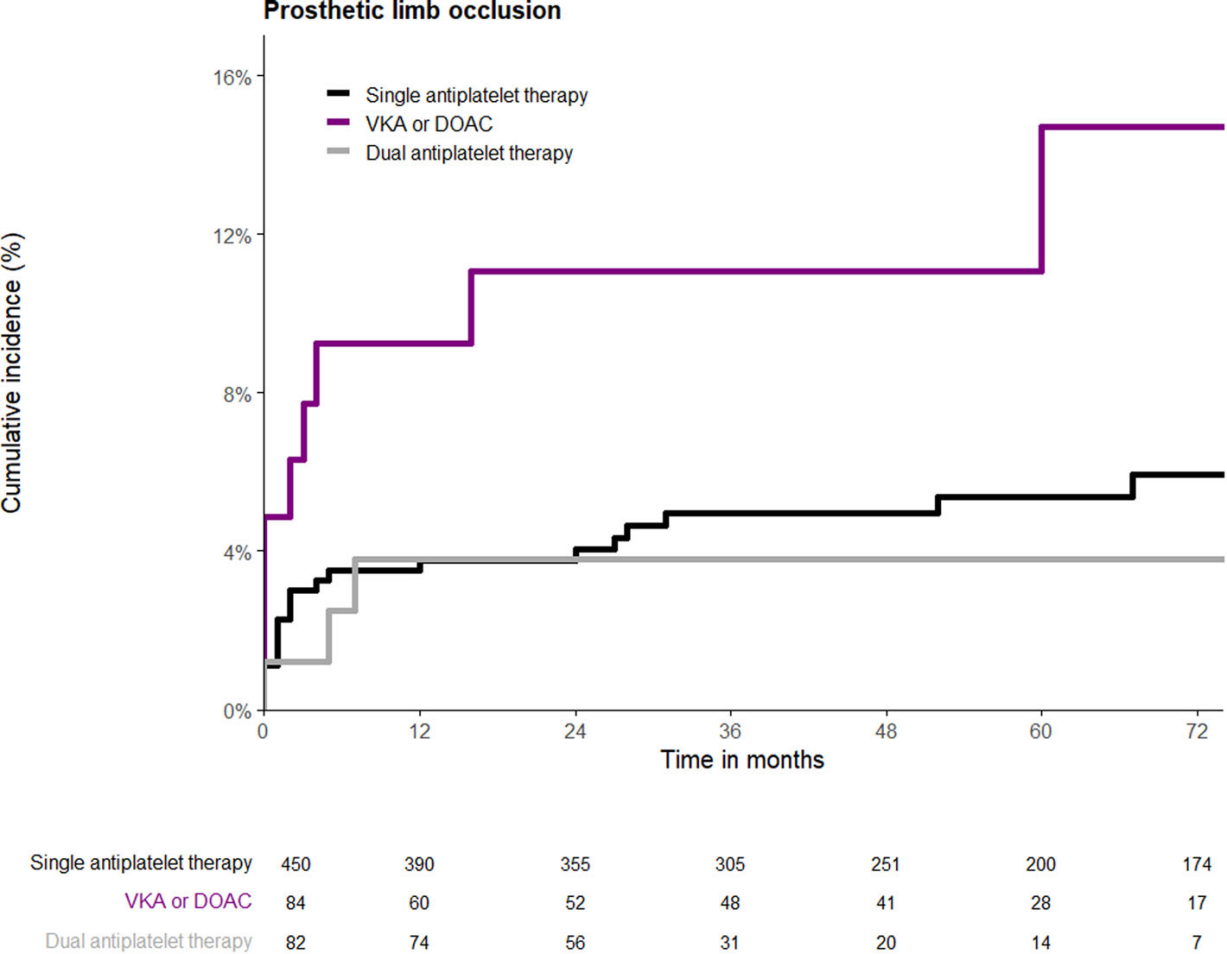
Kaplan–Meier time-to-event analysis for prosthetic limb occlusion based on antithrombotic regimen

Prosthetic limb occlusion occurred in a total of 38 patients (6.2%) during a median follow-up of 4 years (IQR 5 years). The incidence of prosthetic limb occlusion did not change over time. In 33 patients (86.8%) with a prosthetic limb occlusion, there were no predisposing risk factors making them more susceptible for this adverse outcome. Ten (26.3%) prosthetic limb occlusions occurred within the first 30 days after EVAR, and 25 events (65.8%) occurred within the first year following the procedure. Figure 2 shows the KM analysis for prosthetic limb occlusion in the different antithrombotic groups. The log-rank test showed no statistically significant difference (*p* = 0.078) in occurrence of prosthetic limb occlusion between patients receiving SAPT (5.8%), anticoagulants (10.7%), and DAPT (3.7%). Multivariable Cox regression analysis (Table 2) showed that age (HR = 0.96; 95% CI 0.92–0.99; *P* = 0.026) was significantly associated with prosthetic limb occlusion. With increasing age, there is a decrease in the rate of prosthetic limb occlusion, in contrast to the relationship observed between age and occurrence of MACE. Furthermore, in our multivariable analysis, antithrombotic regimen (*p* = 0.017), particularly the use of anticoagulants, showed a significant effect on prosthetic limb occlusion. Patients on anticoagulant therapy showed a significant higher hazard ratio (HR = 3.79, 95% CI 1.46–9.83; *p* = 0.006) implicating that the prosthetic limb occlusion rate in this group of patients is higher than in the reference group of patients using single antiplatelet therapy. We performed a sub-analysis to separately evaluate the effects of VKAs and DOACs on prosthetic limb occlusion, which can be found in Supplemental Table 2. The results were similar to our primary analysis; however, the DOAC subgroup was too small to draw definitive conclusions. Additionally, we performed a multivariable Cox regression analysis including endograft type, among the previously mentioned covariates. Endograft type was not significantly associated with prosthetic limb occlusion, while treatment with anticoagulants remained significantly associated with a higher rate of prosthetic limb occlusion. Out of the total 616 patients 50 (8.3%) underwent FEVAR. No difference was found in the occurrence of prosthetic limb occlusion between patients treated with EVAR compared to FEVAR (*p* = 0.177).

**Fig. 3:**
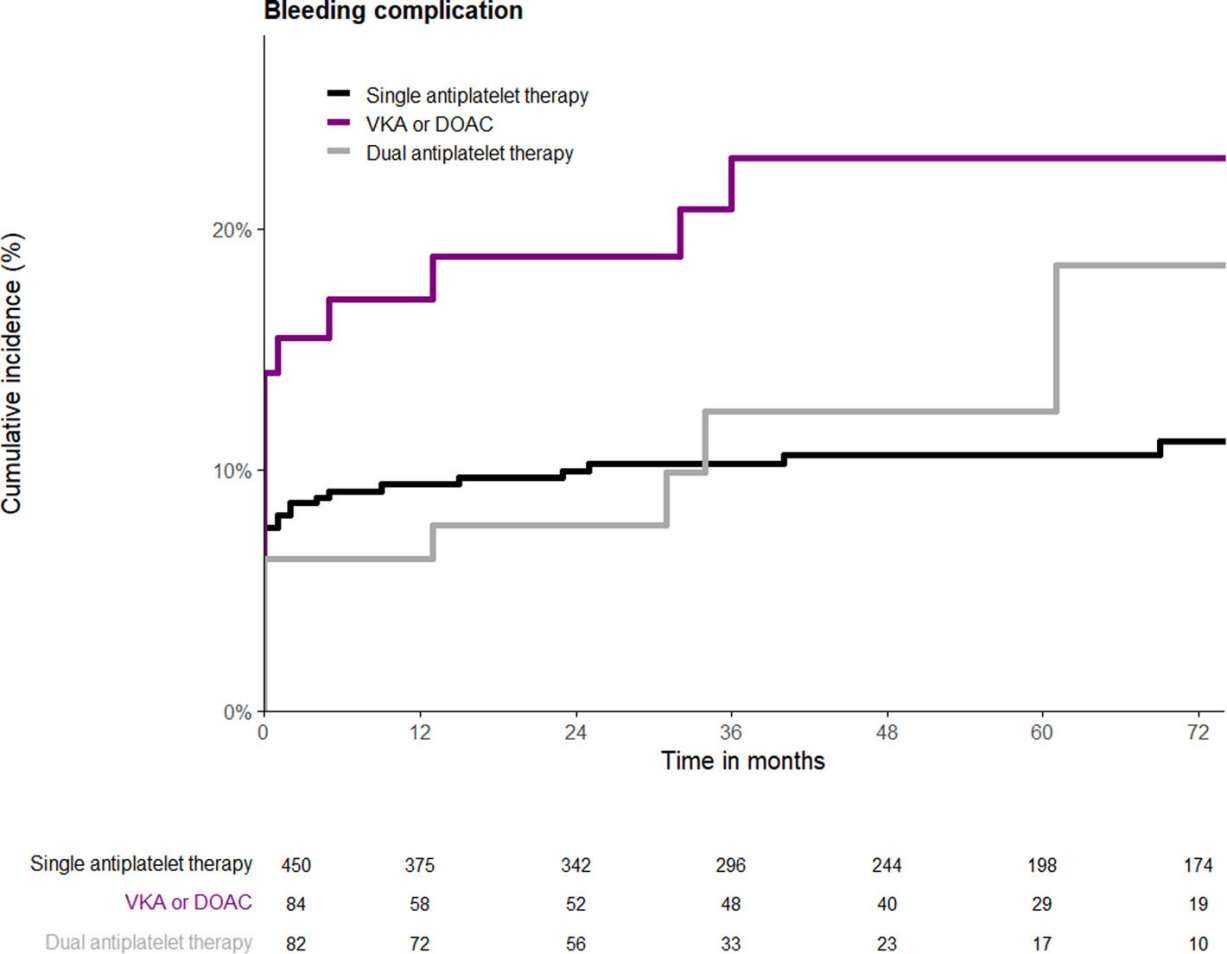
Kaplan–Meier time-to-event analysis for bleeding complication based on antithrombotic regimen

Clinically relevant bleeding complications occurred in a total of 73 patients (11.9%) during a median follow-up of 3 years and 11 months (IQR 5 years and 4 months). A total of 49 (67.1%) clinically relevant bleeding complications occurred within the first 30 days after EVAR. Figure 3 shows the KM analysis for occurrence of a bleeding complication in the different antithrombotic groups. The log-rank test showed no statistically significant difference (*p* = 0.062) in occurrence of bleeding complications between patients receiving SAPT (10.7%), anticoagulants (19.0%), and DAPT (11.0%). In multivariable Cox regression analysis (Table 2), ASA classification (HR = 1.74; 95% CI 1.23–2.46; *p* = 0.002) was significantly associated with bleeding complications. Higher ASA classification showed a higher rate of bleeding complications.

Event rates and multivariable Cox regression analysis for MACE, prosthetic limb occlusion, and bleeding complications within the first 30 days after EVAR can be found in Supplemental Table 1. Additionally, KM and log-rank analyses were performed for occurrence of MACE (Supplemental Fig. 1), prosthetic limb occlusion (Supplemental Fig. 2), and bleeding complications (Supplemental Fig. 3) based on endograft type. There were no statistically significant differences in thrombotic outcomes between patients treated with an Endurant (Medtronic, Santa Rosa, CA), Excluder (W.L. Gore and associates, Flagstaff, AZ), Anaconda (Terumo Aortic, Inchinnan, UK), or other endograft.

## Discussion

In this study, patients using anticoagulants had a more than threefold increased risk of prosthetic limb occlusion compared to patients using single antiplatelet therapy after EVAR. Prosthetic limb occlusions are the third most common reason for readmission after EVAR and usually require reintervention [32, 33]. Anatomical factors, such as tortuosity of the iliac artery, or graft-related factors, such as oversizing, are known risk factors for prosthetic limb occlusion [19, 34–36]. Previous studies have shown that the Anaconda endograft is known for having a relatively higher limb occlusion rate compared to other endografts [37–40]. While our study showed notable variation in prosthetic limb occlusion rates among different endograft types, this variation was not statistically significant. Notably, patients using anticoagulants displayed the highest rate of limb occlusion in our study, despite the Anaconda endograft being most frequently used in the DAPT group.

The influence of antithrombotic regimen on the occurrence of limb occlusion after EVAR is not well investigated. A study of Russell et al. showed that escalation of antithrombotic therapy in case of prosthetic graft thrombus, either by the addition of an antiplatelet agent or by the addition of a DOAC or VKA, was associated with regression or prevention of progression of the graft thrombus [41] However, Oliveira et al. showed that mural thrombus formation in the endograft itself is not associated with the occurrence of thrombotic events, including limb occlusion, over time [42]. Previous research did show younger age to be associated with the occurrence of prosthetic limb occlusions, which was also observed in this study [43].

In contrast to our findings on prosthetic limb occlusion, antithrombotic treatment regimen was not significantly associated with the occurrence of MACE in our study. This is in line with prior research indicating that the influence of antithrombotic therapy on MACE is less pronounced compared to its effects on limb-related events [44]. Older age and significant comorbidities, as reflected by higher ASA-classifications, were identified as predictors of MACE in our cohort, which is consistent with earlier findings [45]. Although we observed no significant associations between antithrombotic treatment regimen or age and clinically relevant bleeding complications, higher ASA classification was associated with an elevated risk of bleeding. Our study is most likely underpowered to detect a significant difference in clinically relevant bleeding complications, as it is well-established that intensifying antithrombotic therapy generally increases the risk of bleeding complications [25, 28, 44]. Patient factors such as age, comorbidities and overall condition are also contributors for an elevated risk of bleeding complications [46].

A potential explanation for our findings is the substantial impact of EVAR procedures on platelet count and activity, which supports the hypothesis that platelet inhibition may be more effective than targeting the coagulation cascade in preventing prosthetic limb occlusions [47, 48]. To explore the effectiveness of different antithrombotic regimens randomized controlled trials or large cohort studies are necessary to provide more definitive evidence. Furthermore, recent trials such as VOYAGER PAD and COMPASS have suggested that dual pathway inhibition (the addition of low-dose rivaroxaban to aspirin) may reduce thrombotic events in patients with peripheral artery disease (PAD) and coronary artery disease (CAD) [26, 28]. While these findings are promising, direct application of these results to the EVAR population is not straightforward due to differences in patient characteristics, types of interventions, and associated risks. Therefore, future research should focus on determining which subgroups of patients undergoing EVAR may benefit from DAPT or dual inhibition therapy, as well as identifying the optimal duration and dosing of such therapies.

In our study, use of anticoagulants appeared to be associated with prosthetic limb occlusion. In previous literature, its use is also associated with an increased risk of all-cause mortality, endoleak and reinterventions after EVAR [21, 22, 49, 50]. Therefore it is important to make a critical analysis of benefits and risks in patients with an indication for EVAR who may require prolonged anticoagulation treatment [22]. Furthermore, escalation to anticoagulants might not be the best choice of treatment after prosthetic limb occlusion and when escalation is needed in patients presenting with mural thrombus. The addition of another antiplatelet agent or low-dose rivaroxaban to the antiplatelet regimen might be more beneficial than conversion to VKA or DOAC, although this needs to be confirmed in future research.

A notable limitation of our study is the grouping of patients using VKAs and DOACs into a single category, despite their likely heterogeneity. To address this, we conducted a sub-analysis separating VKAs and DOACs, revealing a significant association between VKA use and prosthetic limb occlusion. Patients on DOACs showed the highest hazard ratio (HR) for limb occlusion, but this difference was not statistically significant due to the small sample size, leading to very wide confidence intervals. Further research is essential to assess the distinct efficacy and safety profiles of VKAs and individual DOACs in the EVAR population. Large, multicenter studies are needed to clarify their roles in preventing thrombotic events, MACE, and bleeding complications after EVAR.

In addition to the challenges posed by grouping anticoagulants, other limitations of this study include its retrospective design, the lack of pharmacogenetic testing in patients using clopidogrel, and the relatively small numbers of participants with thrombotic and bleeding complications. Information on the origin of the thrombotic event, embolic versus thrombotic, is lacking which limits our insights regarding the etiology of the thrombotic events in our population. Furthermore, no information was available on bridging in our study population. Although we found considerable differences in rates of thrombotic and bleeding complications among both the antithrombotic groups and various endograft types, it is plausible that our study lacked sufficient statistical power to detect these clinically relevant differences. Due to the limited number of events and missing data regarding indication for anticoagulant treatment or differences in baseline cardiovascular risk factors in a substantial part of our study population, we were unable to adjust for all potential confounders in our multivariable analysis. Moreover, there was a considerable amount of censored cases in our Kaplan–Meier and multivariable Cox regression analysis, since participants died or switched to another antithrombotic regimen before occurrence of an outcome of interest.

Altogether this means that the data from this study do not justify robust conclusions, but may support a slightly more reluctant approach in prescribing VKAs or DOACs to patients after EVAR. Research on the benefits of different antithrombotic therapy regimens after EVAR are unclear, and more high-quality data are required [21, 50]. Therefore, further research on this topic is necessary, and the findings reported here may be helpful in designing new clinical trials.

## Conclusion

In our study, the use of anticoagulants after EVAR appears to be associated with an increased risk of prosthetic limb occlusion compared to the use of single or dual antiplatelet therapy, while there was no significant difference in occurrence of MACE or bleeding complications between the three antithrombotic groups.

## Supplementary Information

Below is the link to the electronic supplementary material.

Supplementary file1 (TIFF 2344 KB)

Supplementary file2 (TIFF 2344 KB)

Supplementary file3 (TIFF 2344 KB)

Supplementary file4 (DOCX 17 KB)
