# Supplementary figures and images for: Impact of Antithrombotic Therapy on Thrombotic and Bleeding Complications after Elective Endovascular Repair of Abdominal Aortic Aneurysms
Source: Cardiovasc Intervent Radiol. 2025 Jan 16;48(2):157–66. doi: 10.1007/s00270-024-03946-z (PMC11790793; doi:10.1007/s00270-024-03946-z)

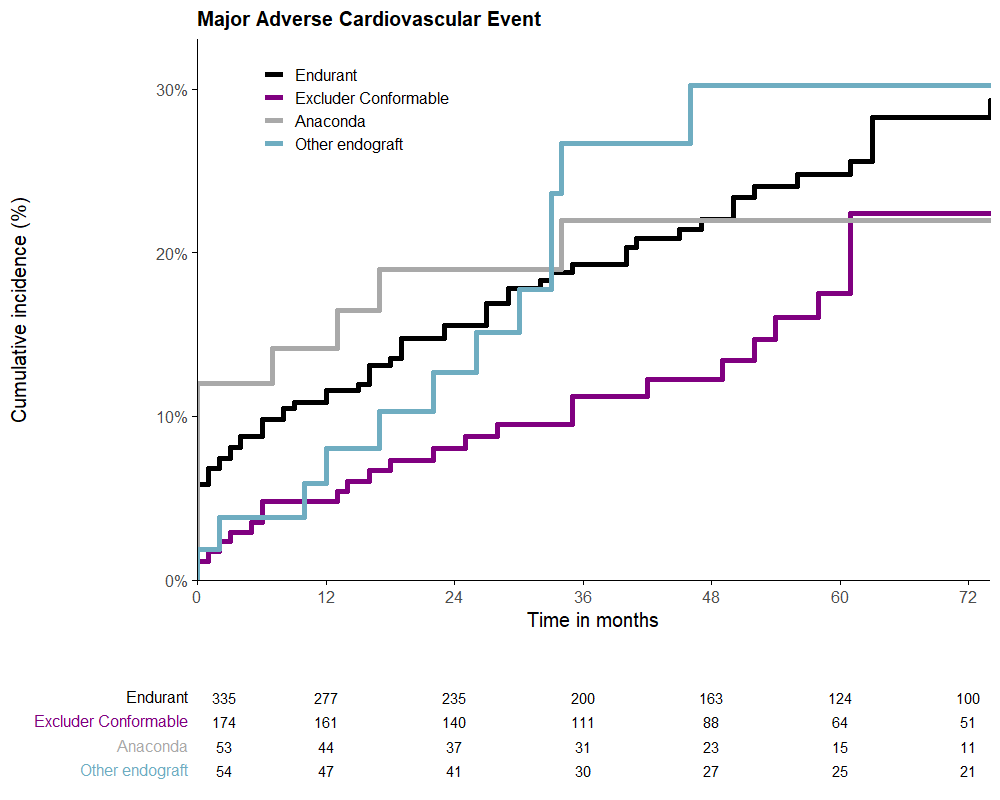

Supplement: Supplementary file 1 — Supplementary file1 (TIFF 2344 KB) [file 270_2024_3946_MOESM1_ESM.tiff]

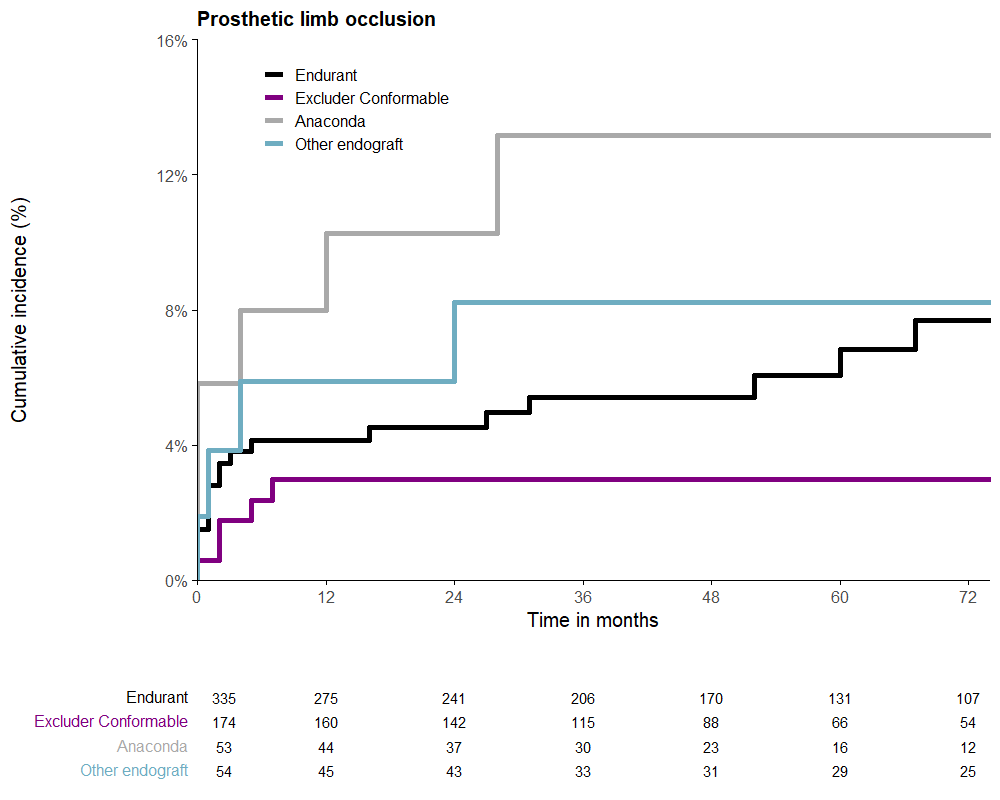

Supplement: Supplementary file 2 — Supplementary file2 (TIFF 2344 KB) [file 270_2024_3946_MOESM2_ESM.tiff]

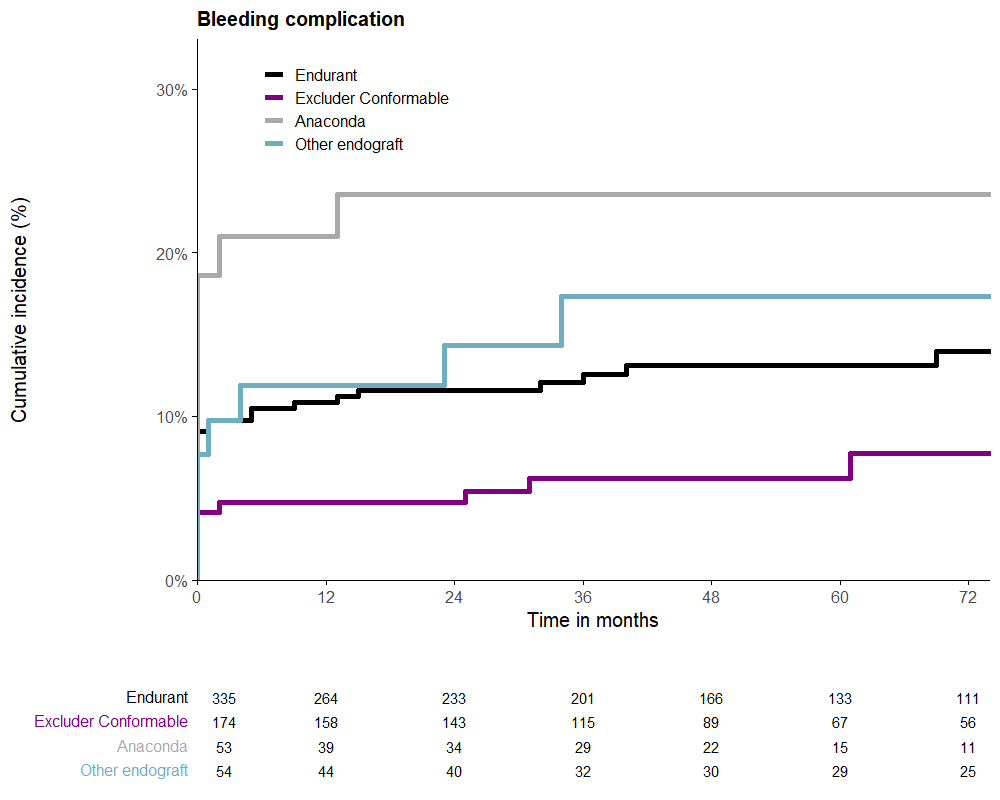

Supplement: Supplementary file 3 — Supplementary file3 (TIFF 2344 KB) [file 270_2024_3946_MOESM3_ESM.tiff]
