# Supplementary material for: Impact of Antithrombotic Therapy on Thrombotic and Bleeding Complications after Elective Endovascular Repair of Abdominal Aortic Aneurysms
Source: Cardiovasc Intervent Radiol. 2025 Jan 16;48(2):157–66. doi: 10.1007/s00270-024-03946-z (PMC11790793; doi:10.1007/s00270-024-03946-z)
Supplement: Supplementary file 4 — Supplementary file4 (DOCX 17 KB) [file 270_2024_3946_MOESM4_ESM.docx]

**Supplemental Table I: Multivariable Cox regression analysis for major adverse cardiovascular events, prosthetic limb occlusion, and bleeding complications within 30 days**

|  | **Major adverse cardiovascular events** | | **Prosthetic limb occlusion** | | **Bleeding complication** | |
| --- | --- | --- | --- | --- | --- | --- |
| **Variable** | **Hazardratio (95% CI)** | **p-value** | **Hazardratio (95% CI)** | **p-value** | **Hazardratio (95% CI)** | **p-value** |
| Age* | 1.03 (0.99-1.08) | 0.176 | 0.96 (0.89-1.04) | 0.316 | 1.01 (0.98-1.05) | 0.460 |
| ASA classification | 4.17 (2.27-7.65) | <0.001 | 0.95 (0.37-2.47) | 0.919 | 1.92 (1.26-2.93) | 0.002 |
| Atrial fibrillation | 1.76 (0.56-5.56) | 0.332 | 1.13 (0.19-6.87) | 0.896 | 0.74 (0.29-1.90) | 0.535 |
| Antithrombotic regimen† |  | 0.636 |  | 0.228 |  | 0.420 |
| Anticoagulants | 0.56 (0.16-1.97) | 0.369 | 4.58 (0.79-26.47) | 0.089 | 1.62 (0.68-3.82) | 0.269 |
| DAPT | 0.78 (0.23-2.62) | 0.683 | 1.17 (0.14-10.18) | 0.885 | 0.77 (0.30-1.97) | 0.583 |

*HR of the clinical event due to 1-year increase in age
†Reference group: single antiplatelet therapy
 Abbreviations: ASA, American Society of Anesthesiologists; DAPT, dual antiplatelet therapy.

Early postoperative outcomes

Within the first 30 days following the EVAR procedure 28 patients (4.5%) experienced MACE and 10 patients (1.6%) experienced a prosthetic limb occlusion. Multivariable Cox regression analysis (Supplemental Table I), adjusted for age, ASA classification, history of atrial fibrillation, and antithrombotic regimen, showed that only ASA classification (HR=4.17; 95% CI 2.27-7.65; p<0.001) was significantly associated with MACE within 30 days postoperative. Higher ASA classification was related to a higher rate of early MACE. The multivariable Cox regression analysis for prosthetic limb occlusion showed that none of the covariates were statistically significant associated with prosthetic limb occlusion within 30 days postoperative. Bleeding complications occurred in 49 patients (8.0%) within 30 days of the procedure. Multivariable Cox regression analysis showed that ASA classification (HR=1.92; 95% CI 1.26-2.93; p=0.002) was significantly associated with bleeding complications within 30 days postoperative. Higher ASA classification was related to a higher rate of bleeding complications.

**Supplemental Table II: Multivariable Cox regression analysis for prosthetic limb occlusion in patients using vitamin-K antagonists, direct oral anticoagulants, or DAPT compared to patients on SAPT**

|  | **Prosthetic limb occlusion** | |
| --- | --- | --- |
| **Variable** | **Hazardratio (95% CI)** | **p-value** |
| Age* | 0.96 (0.92-0.99) | 0.027 |
| ASA classification | 0.98 (0.60-1.61) | 0.933 |
| Atrial fibrillation | 0.46 (0.14-1.46) | 0.185 |
| Antithrombotic regimen† |  | 0.038 |
| Vitamin-K antagonist (N=68) | 3.58 (1.31-9.77) | 0.013 |
| Direct oral anticoagulant (N=16) | 4..87 (0.99-23.77) | 0.050 |
| DAPT | 0.81 (0.24-2.70) | 0.728 |

*HR of the clinical event due to 1-year increase in age
†Reference group: single antiplatelet therapy
 Abbreviations: ASA, American Society of Anesthesiologists; DAPT, dual antiplatelet therapy.
